# Time-to-care metrics in patients with interhospital transfer for mechanical thrombectomy in north-east Germany: Primary telestroke centers in rural areas vs. primary stroke centers in a metropolitan area

**DOI:** 10.3389/fneur.2022.1046564

**Published:** 2023-01-09

**Authors:** Christoph Riegler, Janina R. Behrens, Claudia Gorski, Anselm Angermaier, Stephan Kinze, Ramanan Ganeshan, Andrea Rocco, Alexander Kunz, Tobias J. Müller, Andreas Bitsch, Albert Grüger, Joachim E. Weber, Eberhard Siebert, Kerstin Bollweg, Regina von Rennenberg, Heinrich J. Audebert, Christian H. Nolte, Hebun Erdur

**Affiliations:** ^1^Klinik und Hochschulambulanz Für Neurologie, Charité—Universitätsmedizin Berlin, Berlin, Germany; ^2^Center for Stroke Research Berlin, Charité—Universitätsmedizin Berlin, Berlin, Germany; ^3^NeuroCure Clinical Research Center, Charité and Experimental and Clinical Research Center Charité—Universitätsmedizin Berlin, Berlin, Germany; ^4^Acute Neurological Care for North-East Germany With TeleMedicine Support (ANNOTeM), Berlin, Germany; ^5^Klinik und Poliklinik Für Neurologie, Universitätsmedizin Greifswald, Greifswald, Germany; ^6^BG Klinik Unfallkrankenhaus Berlin, Institut für Telemedizin, Berlin, Germany; ^7^Klinik Für Neurologie, BG Klinik Unfallkrankenhaus Berlin, Berlin, Germany; ^8^Klinik Für Neurologie und Klinische Neuropsychologie, Klinikum Ernst von Bergmann, Potsdam, Germany; ^9^Klinik Für Neurologie, Asklepios Fachklinikum Brandenburg, Brandenburg, Germany; ^10^Klinik Für Neurologie, Universitätsklinikum Ruppin-Brandenburg, Neuruppin, Germany; ^11^Klinik Für Neurologie, Asklepios Fachklinikum Teupitz, Teupitz, Germany; ^12^Klinik Für Neurologie, GLG Martin Gropius Krankenhaus Eberswalde, Eberswalde, Germany; ^13^Berlin Institute of Health, Charité—Universitätsmedizin Berlin, Berlin, Germany; ^14^Institut Für Neuroradiologie, Charité—Universitätsmedizin Berlin, Berlin, Germany; ^15^Corporate Member of Freie Universität Berlin and Humboldt-Universität zu Berlin, Berlin, Germany; ^16^Deutsches Zentrum Für Herz-Kreislaufforschung DZHK, Berlin, Germany

**Keywords:** stroke systems of care, telemedicine, telestroke network, thrombectomy, large vessel occlusions, ischemic stroke, emergency medicine

## Abstract

**Background:**

Mechanical thrombectomy (MT) is highly effective in large vessel occlusion (LVO) stroke. In north-east Germany, many rural hospitals do not have continuous neurological expertise onsite and secondary transport to MT capable comprehensive stroke centers (CSC) is necessary. In metropolitan areas, small hospitals often have neurology departments, but cannot perform MT. Thus, interhospital transport to CSCs is also required. Here, we compare time-to-care metrics and outcomes in patients receiving MT after interhospital transfer from primary stroke centers (PCSs) to CSCs in rural vs. metropolitan areas.

**Methods:**

Patients from ten rural telestroke centers (RTCs) and nine CSCs participated in this study under the quality assurance registry for thrombectomies of the *Acute Neurological care in North-east Germany with TeleMedicine (ANNOTeM)* telestroke network. For the metropolitan area, we included patients admitted to 13 hospitals without thrombectomy capabilities (metropolitan primary stroke centers, MPSCs) and transferred to two CSCs. We compared groups regarding baseline variables, time-to-care metrics, clinical, and technical outcomes.

**Results:**

Between October 2018 and June 2022, 50 patients were transferred from RTCs within the ANNOTeM network and 42 from MPSCs within the Berlin metropolitan area. RTC patients were older (77 vs. 72 yrs, *p* = 0.05) and had more severe strokes (NIHSS 17 vs. 10 pts., *p* < 0.01). In patients with intravenous thrombolysis (IVT; 34.0 and 40.5%, respectively), time from arrival at the primary stroke center to start of IVT was longer in RTCs (65 vs. 37 min, *p* < 0.01). However, RTC patients significantly quicker underwent groin puncture at CSCs (door-to-groin time: 42 vs. 60 min, *p* < 0.01). Despite longer transport distances from RTCs to CSCs (55 vs. 22 km, *p* < 0.001), there was no significant difference of times between arrival at the PSC and groin puncture (210 vs. 208 min, *p* = 0.96). In adjusted analyses, there was no significant difference in clinical and technical outcomes.

**Conclusion:**

Despite considerable differences in the setting of stroke treatment in rural and metropolitan areas, overall time-to-care metrics were similar. Targets of process improvement should be door-to-needle times in RTCs, transfer organization, and door-to-groin times in CSCs wherever such process times are above best-practice models.

## Introduction

**Figure 1:**
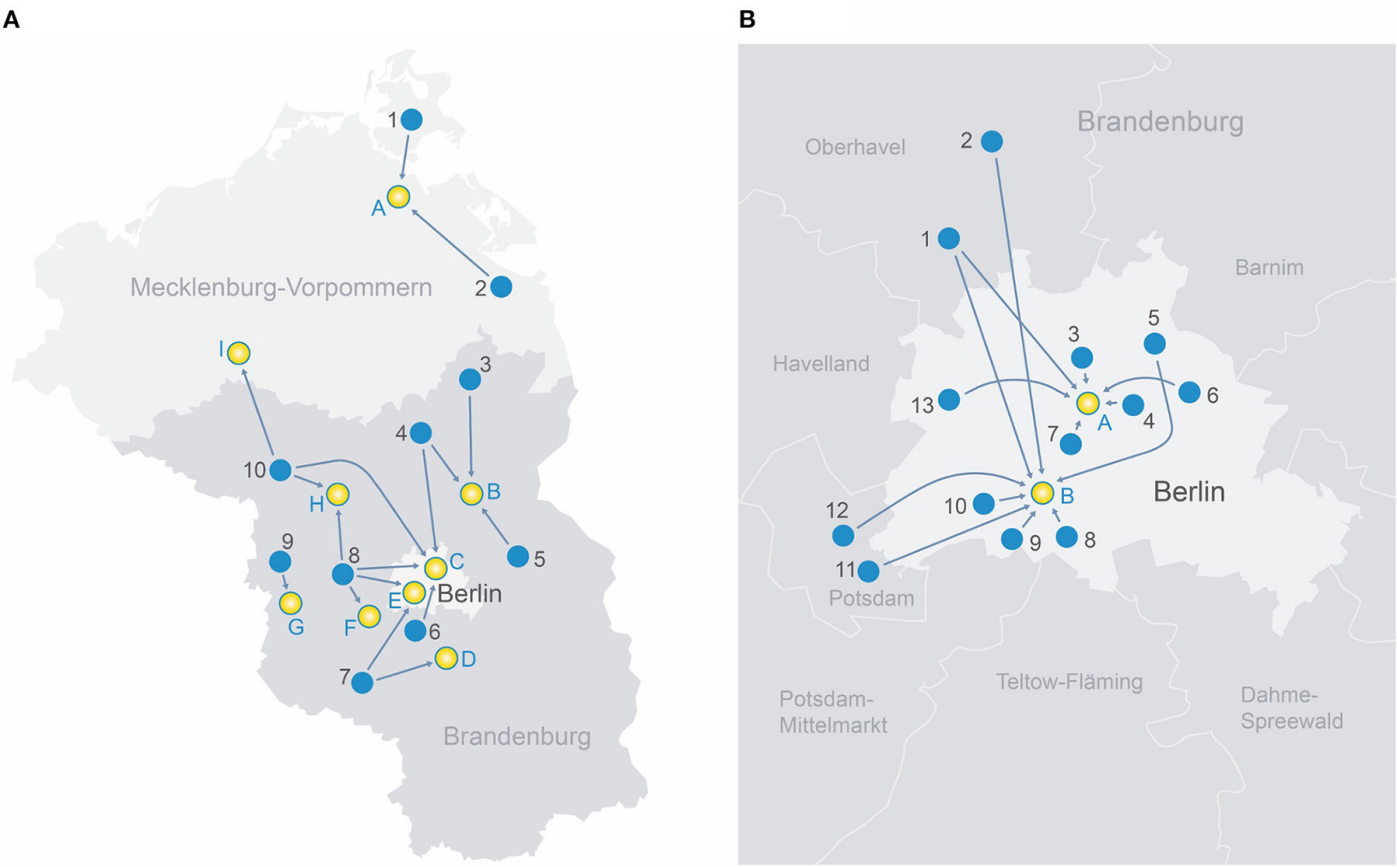
**(A)** Transport routes in the rural region of north-east Germany. 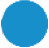
 **Rural telestroke centers** 1. Sana-Krankenhaus Rügen GmbH 2. AMEOS Klinikum Ueckermünde 3. GLG Kreiskrankenhaus Prenzlau 4. Sana Krankenhaus Templin 5. Krankenhaus Märkisch-Oderland GmbH 6. Evangelisches Krankenhaus Ludwigsfelde-Teltow 7. KMG Klinikum Luckenwalde 8. Havelland Kliniken Gmbh Nauen 9. Havelland Klinik Gmbh Rathenow 10. KMG Klinikum Kyritz 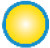
 **Comprehensive stroke centers** A. Universitätsklinikum Greifswald B. GLG Martin Gropius Krankenhaus Eberswalde C. BG Klinikum Unfallkrankenhaus Berlin D. Asklepios Fachklinikum Teupitz E. Charité Campus Benjamin Franklin Berlin F. Klinikum Ernst von Bergmann Potsdam G. Asklepios Fachklinik Brandenburg H. Universitätsklinikum Ruppin-Brandenburg I. MEDICLIN Krankenhaus Plau am See **(B)** Transport routes in the metropolitan area of Berlin, Germany. 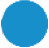
 **Metropolitan primary stroke centers** 1. Oberhavel Kliniken GmbH—Klinik Hennigsdorf 2. Oberhavel Klinik Oranienburg 3. Bundeswehrkrankenhaus Berlin 4. Alexianer St. Hedwig-Krankenhaus Berlin 5. Park-Klinik Weißensee Berlin 6. KEH Evangelisches Krankenhaus Königin Elisabeth Herzberge Berlin 7. Evangelische Elisabeth Klinik Berlin 8. Krankenhaus Bethel Berlin 9. Helios Klinikum Emil von Behring Berlin 10. Krankenhaus Waldfriede Berlin 11. Klinikum Ernst von Bergmann Potsdam 12. Alexianer St. Josefs-Krankenhaus 13. Klinikum Spandau-Vivantes Berlin 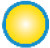
 **Comprehensive stroke centers** A. Charité Campus Mitte Berlin B. Charité Campus Benjamin Franklin Berlin.

In Germany, shortage of specialized physicians is an increasing challenge to the healthcare system and is particularly pronounced in rural areas. This disparity is illustrated by a comparison between the rural area of north-east Germany and the metropolitan area of Berlin, showing the highest gap in health service quality nationwide. While Berlin has one doctor for 149 inhabitants, the federal state of Brandenburg—surrounding Berlin (see Figure 1)—provides only one doctor for 246 inhabitants (1). In Germany and other countries, studies demonstrated disparities in stroke outcome between patients treated in rural vs. metropolitan areas (2–5). These disparities may result from multiple factors like longer distances and transport times or expert shortage or different processes of acute management in emergency rooms (2–5). To overcome these differences, telestroke networks were implemented to provide expert guidance for rural telestroke centers (RTCs) without (continuous) neurological expertise (2, 6, 7). Since 2002, more than 20 stroke networks were established in Germany and significantly improved local stroke care based on standardized operating procedures (SOP) and evidence-based acute treatments (6, 8–11). Network support led to implementation of treatment-relevant algorithms in rural hospitals, such as CT angiography guided screening for large vessel occlusions (LVO) (12). In the case of specific LVO detection, guidelines recommend mechanical thrombectomy (MT) as standard of care (13–15). Although MT proved to be efficient up to 24 h from stroke onset in selected patients, outcomes after MT are still highly time dependent and ideally MT should be initiated as early as possible after symptom onset (16, 17). While telestroke networks enable RTCs to rapidly initiate intravenous thrombolysis (IVT) on site, LVO stroke patients still need transportation to comprehensive stroke centers (CSCs) before neuro-interventional therapy can be initiated.

Interhospital transport is associated with treatment delays (e.g., longer times from symptom onset to groin puncture), hence a lower odds of good clinical outcome has been reported in previous studies (2, 18–20). While there is good evidence on shorter treatment delays and better clinical outcomes in patients who receive IVT in rural hospitals with telemedical network support (6, 8–10, 21), real-life studies comparing process parameters and outcomes after MT in rural areas with telemedical support with metropolitan areas remain sparse (22).

To address this matter, we compared real-world data of MT treated patients within the *Acute neurological care in north-east Germany with telemedicine support (ANNOTeM)* network, supporting acute stroke treatment in rural telestroke hospitals in the north-eastern federal states of Germany (23) to those admitted to primary stroke centers (PSCs) in the metropolitan area of Berlin.

## Methods

### Study population

We compared mechanical thrombectomy candidates transferred to CSCs from two different types of PSCs. The first group of patients was treated in rural telestroke centers (RTCs), working within a local telestroke network (described in detail below). The second group of patients was transferred from metropolitan primary stroke centers (MPSCs) in the area of Berlin. Patient data from 10/2018 to 06/2022 were gathered inside two large multicenter observational registry studies, both assessing the quality of processes as well as technical and clinical outcomes of MT-treated patients. In-hospital strokes were not included into the presented study.

### ANNOTeM network

The ANNOTeM network (23) consists of three comprehensive stroke centers (Charité—Universitätsmedizin Berlin, Universitätsmedizin Greifswald and BG Unfallkrankenhaus Berlin) and provides a managed care system including a 24/7 telemedicine service for 11 rural hospitals in the north-eastern federal states of Germany (Mecklenburg-Western Pomerania, Brandenburg, and Saxony-Anhalt). The network provides audiovisual counseling conducted by an experienced stroke neurologist, guiding the local team through diagnostic and treatment processes (including intravenous thrombolysis for ischemic stroke). In patients with indication for mechanical thrombectomy, the teleneurologist organizes the interhospital transfer process from activation of the emergency medical transport service, allocating the patient to the nearest interventional center and pre-notification of the intervention team at the CSCs.

### German stroke registry

The control group consists of individuals with large or medium vessel occlusion stroke, admitted to hospitals without thrombectomy capabilities in the metropolitan area of Berlin, Germany. These patients were then transported to two large CSCs, both part of the *German Stroke Registry (GSR-MT)*, a national, prospective, multicenter observational registry. As detailed in the study protocol, the GSR-MT includes all patients admitted to its participating centers with ischemic stroke, aged ≥18 years in which mechanical thrombectomy (MT) is initiated (24). All GSR patients who fulfilled the above-mentioned criteria and received MT at the two CSCs were included in the analysis (including seven patients with initial presentation at a hospital without neurology department).

### Variables

We compared both groups regarding baseline variables, differences in treatment modality (e.g., IVT and anesthesia), process times as well as clinical and technical outcomes. Initial stroke severity was assessed using the National Institutes of Health Stroke Scale (NIHSS). To assess potential imbalances regarding vessel occlusion site, we dichotomized patients into proximal large vessel occlusion (internal carotid artery (ICA), proximal and distal M1 segment of the middle cerebral artery (MCA), basilar artery) and medium vessel occlusions [MCA M2 segment, vertebral artery (VA), anterior cerebral artery (ACA), posterior cerebral artery (PCA)]. Technical reperfusion outcome was assessed using the modified Thrombolysis in Cerebral Infarction scale (mTICI). As clinical outcome, we chose disability at discharge, using the modified Rankin Scale (mRS). We defined in-hospital death and any intracranial hemorrhage (ICH) as safety outcomes.

### Statistical analyses

Continuous baseline variables and treatment times are presented as median [IQR], dichotomous variables as absolute numbers and percentage. Comparisons regarding distribution between groups were performed by Kruskal–Wallis- and Chi-Square test. Binary logistic regression analyses were carried out to assess the impact of hospital admission site (metropolitan vs. rural) on clinical and technical outcomes. Odds Ratios (ORs) for clinical, technical and safety outcomes were adjusted for age, sex, stroke severity (NIHSS on admission), vessel occlusion site (posterior vs. anterior) and IVT. To address the potential influence of repeated imaging at the CSC on time-to-care metrics, we conducted a sensitivity analysis comparing door-to-groin time separately for patients with and without repeated imaging at the CSC. All analyses were carried out using IBM SPSS Statistics for Windows, Version 27.0. Armonk, NY: IBM Corp.

## Results

### Study population

Our study consisted of 92 individuals who received MT between 10/2018 and 06/2022 at one of the participating centers. Fifty individuals (54%) were primarily admitted at ten different rural telestroke centers within the ANNOTeM network and 42 patients (46%) presented at one of 13 metropolitan primary stroke centers in the area of Berlin. In order to receive MT, patients from RTCs were transferred to nine different CSCs. Patients from MPSCs were transferred to one of the two university CSCs in Berlin. Figure 1 depicts transport routes between PSCs and CSCs in rural and metropolitan areas, respectively (Figure 1A: ANNOTeM network in north-east Germany; Figure 1B: Metropolitan region of Berlin).

### Baseline variables

We found several differences in baseline variables between the two groups. RTC patients were older (median [IQR]: 77 [68–83] vs. 72 [61–80] years, *p* = 0.054), had more severe strokes (median NIHSS [IQR]: 17 [13–21] vs. 10 [7–18], *p* < 0.01) and tended to have more proximal LVO and less medium vessel occlusions. For exact distribution of vessel occlusion site, see Supplementary Table 1. There were no differences in the distribution of sex, pre-stroke functional independency, baseline living status, oral anticoagulation, and witnessed onset of stroke. Details are reported in Table 1.

**Table 1:** Demographic and baseline variables.

	**Rural telestroke centers (*n* = 50)**	**Metropolitan primary stroke centers (*n* = 42)**	***p***
Age (years)—median [IQR]	**77** [68–83]	**72** [61–80]	**0.054**
Female sex—*n* (%)	25/50 **(50.0)**	22/42 **(52.4)**	0.82
prestroke mRS ≤ 2—*n* (%)	34/41 **(82.9)**	36/42 **(85.7)**	0.73
NIHSS at admission—median [IQR]	**17** (13–21)	**10** [7–18]	**< 0.01**
Living Status at baseline			0.23
- Home—*n* (%)	29/37 **(78.4)**	37/42 **(88.1)**	
- Nursing at home—*n* (%)	5/37 **(13.5)**	4/42 **(9.5)**	
- Nursing home—*n* (%)	3/37 **(8.1)**	1/42 (**2.4)**	
Anticoagulation—*n* (%)	9/50 **(18.0)**	6/42 **(14.3)**	0.63
Medium vessel occlusion (MCA M2, PCA, VA, ACA)—*n* (%)	9/49 **(18.4)**	13/42 **(31.0)**	0.16
Occlusion in the posterior circulation—*n* (%)	6/50 **(13.7)**	7/42 **(16.7)**	0.52

Chi^2^ for binary variables, Kruskal-Wallis for ordinal and linear variables. The bold values indicated the median and percentage respectively. The bolded *p*-values indicate the significant or borderline-significant results.

### Time-to-care metrics

When focusing on acute treatment processes, the groups differed in several points: First, all but two RTC patients (48/50, 96.0%) were treated at a primary hospital without neurology department (telestroke support only). In contrast, the majority of MPSC patients were treated by a hospital with neurologists on site (35/42, 83.3%), and only 7/42 (16.7%) at a primary hospital without neurology department (*p* < 0.001). While all RTC patients received CT with angiography, six of the above mentioned seven MPSC patients had only plain CT (*p* < 0.01). The transport distances between primary hospitals and CSCs were longer in RTC patients (52 vs. 22 km, *p* < 0.001, see also Figure 1). RTC patients more often received repeated CT-imaging at the CSC before MT was initiated (56.0 vs. 33.3%, *p* = 0.03). Repeated imaging was not associated with time from last scan to arrival at the CSC (OR 0.90 [0.72–1.13] per +30 minutes, *p* = 0.37). Rates of IVT and modality of anesthesia did not differ between groups (for exact values see Table 2).

**Table 2:** Time-to-care metrics.

	**Rural telestroke centers (*n* = 50)**	**Metropolitan primary stroke centers (*n* = 42)**	***p***
Primary hospital without neurology department—*n* (%)	48/50 **(96.0)**	7/42 **(16.7)**	**< 0.001**
Only plain CT in primary hospital—*n* (%)	**0**	6/42 **(14.3)**	**< 0.01**
Intravenous thrombolysis (IVT)—*n* (%)	17/50 **(34.0)**	17/42 **(40.5)**	0.52
Transport distance (km)—median [IQR]^*^	**52** (47–70)	**22** (21,22)	**< 0.001**
Repeated imaging in interventional hospital—*n* (%)	28/50 **(56.0)**	14/42 **(33.3)**	**0.03**
Anesthesia—*n* (%)			0.92
- General	34/49 **(69.4)**	27/40 **(64.3)**	
- Conscious sedation	14/49 **(28.6)**	11/40 **(26.2)**	
- Switch from local to general	1/49 **(2.0)**	2/40 **(4.8)**	
Witnessed onset of stroke—*n* (%)	21/50 **(42.0)**	21/42 (**50.0)**	0.44
Symptom onset to arrival at PSC (minutes)—median [IQR]	**70** [60–120]	**74** [50–146]	0.80
Arrival at PSC to start of IVT **(door-to-needle;** minutes)—median [IQR]	**65** [43–78]	**37** [25–51]	**< 0.01**
Arrival at PSC to arrival at CSC (minutes)—median [IQR]	**161** [148–204]	**150** [123–226]	0.10
Arrival at CSC to arrival at angiography suite (minutes)—median [IQR]	**28** [10–41]	**25** [11–68]	0.28
Arrival at angiography suite to groin puncture (minutes)—median IQR	**15** [10–20]	**25** [16–42]	**< 0.001**
Arrival at CSC to groin puncture **(door-to-groin)** (minutes)—median [IQR]	**42** [21–64]	**60** [38–90]	**< 0.01**
Arrival at PSC to groin puncture (minutes)—median [IQR]	**210** [182–251]	**208** [180–285]	0.96
Imaging at the PSC to groin puncture (minutes)—median [IQR]	**170** [153–226]	**175** [111–214]	0.34
Symptom **onset to groin** puncture (minutes)—median [IQR]	**270** [245–335]	**300** [250–543]	0.41

Chi^2^ for binary variables, Kruskal-Wallis for ordinal and linear variables.

^*^Transport distances of patients transported via helicopter (2/50) were excluded from calculation. PSC, primary stroke center; CSC, comprehensive stroke center. The bold values indicated the median and percentage respectively. The bolded *p*-values indicate the significant or borderline-significant results.

Time from symptom onset to admission in the primary (non-interventional) hospital was similar for RTC and MPSC patients with a median of 70 min. The time from hospital arrival to the initiation of IVT (door-to-needle) was significantly longer in RTCs (65 [43–78] vs. 37 [25–51] min, *p* < 0.01). In contrast, the time from arrival at the CSC to groin puncture was significantly shorter in RTC patients (42 [21–64] vs. 60 [38–90] min, *p* < 0.01). This difference was consistent for patients with and without repeated imaging (50 vs. 91 min; 25 vs. 48 min, *p* < 0.01, respectively).

Splitting door-to-groin time in two parts, the delay between arrival at the CSC and arrival at the angio-suite was similar in both groups (28 [10–41] vs. 25 [11–68] min, *p* = 0.28). The time from arrival at the angio-suite to groin puncture, however, was significantly shorter for patients from RTCs (15 [10–20] vs. 25 [16–42] min, *p* < 0.001)

There was no significant difference in the time interval from PSC admission to arrival at the CSC (161 [148–204] vs. 150 [123–226] min, *p* = 0.10). Measuring the whole process from arrival at the PSCs to groin puncture, treatment times did not differ (210 [182–251] vs. 208 [180–285] min, *p* = 0.96). For further time metrics and exact numbers (see Table 2).

### Outcomes

In univariable analysis, patients treated in the metropolitan region had a higher rate of functional independency at discharge (OR_mR ≤ 2_ = 6.39 [1.92–21.23], *p* < 0.01) and higher odds for surviving without severe disability (OR_mRS ≤ 3_ = 3.17 [1.30–7.69], *p* = 0.01). However, after adjustment for age, sex, NIHSS, vessel occlusion site (posterior vs. anterior) and intravenous thrombolysis, no significant difference in clinical outcomes could be found (aOR_mRS ≤ 2_ = 3.16 [0.83–11.98], *p* = 0.09; aOR_mRS ≤ 3_ = 1.80 [0.59–5.46], *p* = 0.30). Reperfusion rates as well as the rate of intracranial hemorrhage and in-hospital death were similar for patients from rural and metropolitan areas. For exact numbers, see Table 3.

**Table 3:** Outcomes.

**Outcome**	**Rural telestroke centers (*n* = 50)**	**Metropolitan primary stroke centers (*n* = 42)**	**OR [95%-CI]**	***p***	**aOR [95%-CI]**	***p***
mRS ≤ 2 at DC—*n* (%)	4/50 **(8.0)**	15/42 **(35.7)**	6.39 [1.92–21.23]	**< 0.01**	3.16^*^ [0.83–11.98]	0.09
mRS ≤ 3 at DC—*n* (%)	12/50 **(24.0)**	21/42 **(50.0)**	3.17 [1.30–7.69]	**0.01**	1.80^*^ [0.59–5.46]	0.30
Successful reperfusion [mTICI 2b/3—*n* (%)]	38/46 **(82.6)**	37/41 **(90.2)**	1.95 [0.54–7.02]	0.31	3.97^*^ [0.62–25.57]	0.15
Complete reperfusion [mTICI 3—*n* (%)]	26/46 **(56.5)**	26/41 **(63.4)**	1.33 [0.56–3.16]	0.51	0.99^*^ [0.36–2.73]	0.99
Death during hospital stay—*n* (%)	15/50 **(30.0)**	8/42 **(19.0)**	0.55 [0.21–1.46]	0.23	1.17^*^ [0.32–4.27]	0.81
Any intracranial bleeding in follow-up imaging—*n* (%)	10/50 **(20.0)**	10/42 **(23.8)**	1.25 [0.46–3.37]	0.66	1.46^*^ [0.41–5.16]	0.56

^*^Adjusted for age, sex, thrombolysis, NIHSS at admission, vessel occlusion site (posterior vs. anterior). The bold values indicated the median and percentage respectively. The bolded *p*-values indicate the significant or borderline-significant results.

## Discussion

In this study, we report the results of 92 MT-eligible stroke patients who were initially treated at a hospital without MT capabilities and subsequently transported to a CSC. We compared patients treated in rural hospitals inside a telestroke network with patients presenting at metropolitan primary stroke centers. With focus on time-to-care metrics, we report three main findings: First, the administration of IVT was performed significantly faster in the metropolitan hospitals. Secondly, the delay from arrival at the CSC to groin puncture was shorter in patients from rural telestroke centers. Thirdly, however, the whole treatment time from arrival at primary hospitals to groin puncture did not differ significantly between both groups.

Epidemiological investigations in the U.S., Australia, Poland, and Nigeria showed marked discrepancies of stroke treatment procedures and outcome, linked to stroke patients' place of residence. The disparities of rural (without telestroke network support) compared to urban areas were mainly related to a knowledge gap and different distances/transport times (2–5, 18, 25).

In line with these findings, our study showed longer door-to-needle times and longer transport distances in rural areas. The longer delay from patient arrival to IVT initiation in the RTCs may be due less awareness and experience compared to metropolitan hospitals in Berlin, where the most of which had own neurology departments with expertise on site, higher annual IVT numbers and longer experience with IVT treatments. In contrast, IVT was administered only occasionally in the RTCs before implementation of the ANNOTeM telestroke network. Our data generally reflect findings from other telestroke networks, where achieving similar door-to-needle times as in experienced stroke centers has been a struggle (26–28). However, years of continuous education and training in telestroke networks, including stroke certificates or on-site staff training have shown to be effective in reducing door-to-needle times (11, 26). Such measures are part of the quality management in the ANNOTeM network, but it may take longer to produce greater effects as the network was founded in 2017.

At the CSCs, the time from admission to start of the interventional therapy (door-to-groin) was faster in RTC patients (42 vs. 60 min). These door-to-groin times were similar to those in the RACECAT trial in Spain, in which half of the patients received primary treatment in a rural hospital and network-coordinated transport to a CSC (29). The short door-to-groin times may be due to telestroke network coordinated pre-notification of the CSC with digital transfer of CT scans and earlier activation of interventional and anesthesia teams. In MPSC patients, CSCs were also pre-notified. However, there was no standardized process for informing the different members of the team during the study period. By now, a standard operating procedure was implemented at the two university CSCs in order to reduce treatment delays. It might be possible that longer door-to-groin times in the metropolitan patients of our study might also be a consequence of the lower NIHSS scores and the higher frequency of distal vessel occlusion, making interdisciplinary decision processes for/against MT more complex and time consuming. However, assuming the time from arrival at the CSC to arrival at the angio-suite as a proxy for the decision process, we did not find a significant difference that could support this notion. The difference in time from arrival at the angio-suite to groin puncture on the contrary suggests, that the delay in metropolitan patients might rather be due to a longer latency for the interventional team to arrive.

Interestingly, the rate of repeated imaging in the CSC was significantly higher in rural hospital patients. Because of the clinical presentation with higher NIHSS at CSC arrival, MT may have been re-considered in RTC patients more often (screening for secondary hemorrhage or large infarct demarcation). However, we do not have center-specific information about the rationale for repeated imaging and our data show a substantial heterogeneity across centers, making it difficult to address this question. A lower rate of repeated imaging may be a target of process improvement in CSCs. Regarding the paradoxon of a higher rate of repeated imaging and still shorter admission to groin delay in RTC patients, a sensitivity analysis showed that these patients had a shorter door-to-groin time with and without repeated imaging (see results section).

The key process parameters “symptom onset to groin puncture” (270 vs. 300 min) and “arrival at the primary hospital to groin puncture” (210 vs. 208 min) were not longer in patients from rural hospitals—despite significantly longer transport distances (52 vs. 22 kilometers; see Figures 1A, B). This may indicate a fast organization of interhospital transfers in RTC patients, showing the potential benefits of telestroke networks.

In a recently published study on mechanical thrombectomy in patients transferred from rural hospitals in south-east Germany, the median transport distance and even the time from arrival in the primary stroke centers to groin puncture were similar as in our study (30). This may suggest representativeness of our results for time-to-care metrics in rural areas in Germany.

With a view on demographic data (see Table 1), the presented groups of patients differ significantly in stroke severity, age, and site of vessel occlusion. This may be explained by a distribution effect: In the Berlin metropolitan area, there are far more MT capable stroke centers, allowing a direct transfer to a CSC whenever clinical symptoms (e.g., high NIHSS) suggest LVO. In rural areas, on the other hand, a direct transfer to the nearest CSC is usually not possible due to the large distances and bottlenecks in ambulance care.

### Limitations

This study reports results from a relatively small patient sample with patients from 10 rural hospitals participating in the ANNOTeM network which comprise only a little part of many more existing hospitals in north-east Germany (38 hospitals in Mecklenburg-Western Pomerania and 61 in Brandenburg) (31). Thus, we cannot exclude a selection bias. Further, while patients from rural hospitals were treated at nine different CSCs, we analyzed only two CSCs in the metropolitan patients. Thus, local treatment algorithms may differ from other interventional centers in the metropolitan area, which limits the representativeness of our findings. Due to our small sample size and the large number of participating PSCs, we were not able to account for the clustering effect of centers.

There are substantial differences in age, stroke severity and vessel occlusion site between rural and metropolitan patients. This does not only influence clinical and technical outcomes but may also have substantial impact on time-to-care metrics, which we were not able to account for in our analyses.

In our study, we only included patients that received MT, but did not gather data of stroke patients with large or medium vessel occlusion that were transferred, but not thrombectomized. Thus, we were not able to assess whether the actual frequency of MT performance differs between rural and metropolitan patients with MT-eligible vessel occlusion.

Regarding clinical outcome, we only have data on mRS at discharge. Assessing the mRS for hospitalized patients may overpredict functional independency, since some patients who do not require assistance in hospital may not be completely independent at home. Besides, due to the above-mentioned differences in age and stroke severity, a comparison of clinical outcomes is very limited.

Most of the participating PSCs were not capable of performing CT perfusion scans. Thus, we cannot exclude, that rural and metropolitan patients in the late time window had a different distribution of core/penumbra mismatch and consequently a different recovery prognosis.

We were not able to calculate exact transport times since we lacked data of the exact start of interhospital transport (door-out-times). Another aspect in terms of data quality is that transport modalities involving the use of helicopters were only documented for two air transported rural patients. For all other patients, we assume an ambulance transport. However, since we do not have original transport documents, we cannot validate this assumption.

## Conclusion

Regarding the overall time-to-care metrics in patients receiving MT after interhospital transfer (e.g., symptom onset to groin puncture), the process quality seemed to be similar in rural and metropolitan areas. Targets of process improvement may be door-to-needle times in RTCs, transfer organization and door-to-groin times in CSCs wherever such process times are above best-practice models. We suggest the implementation of standard operation procedures (SOPs) to organize interhospital transport of MT-eligible patients in metropolitan areas and initiated this concept at our two university CSCs.

## Data availability statement

The datasets analyzed for this study are available from the authors on reasonable request.

## Author contributions

CR, JRB, and HE were responsible for the study concept, gathered data, performed statistical analyses, and drafted the manuscript. CG, AA, SK, RG, AR, AK, TJM, AB, AG, JEW, ES, KB, RR, HJA, and CHN collected data and critically reviewed the manuscript. All authors contributed to the article and approved the submitted version.
