# Supplementary material for: Time-to-care metrics in patients with interhospital transfer for mechanical thrombectomy in north-east Germany: Primary telestroke centers in rural areas vs. primary stroke centers in a metropolitan area
Source: Front Neurol. 2023 Jan 9;13:1046564. doi: 10.3389/fneur.2022.1046564 (PMC9868735; doi:10.3389/fneur.2022.1046564)
Supplement: Supplementary file 1 [file Table_1.docx]

**Table S1: Distribution of occlusion site in both groups**

|  | **Rural telestroke centers (n=50)** | **Metropolitan primary stroke centers (n=42)** | p |
| --- | --- | --- | --- |
| Location of LVO on CT-angiography – n (%)     - Basilar artery      - Vertebral artery      - Posterior cerebral artery      - Anterior cerebral artery      - Carotid-non-T intracranial      - Carotid-T      - Carotid non-T extracranial      - Proximal MCA M1      - Distal MCA M1      - MCA M2 | 3/49 **(6.1)**    **0**    3/49 **(6.1)**    1/49 **(2.0)**    1/49 **(2.0)**    3/49 **(6.1)**    2/49 **4.1)**    19/49 **(38.8)**    12/49 **(24.5)**    5/49 (**10.2)** | 3/42 **(7.1)**    3/42 **(7.1)**    1/42 **(2.4)**    2/42 **(4.8)**    4/42 **(9.5)**    **0**    4/42 **(9.5)**    10/42 (**23.8)**    8/42 **(19.0)**    7/42 **(16.7)** | 0.11 |
| Medium vessel occlusion (MCA  M2, PCA, VA, ACA)—n (%) | 9/49 **(18.4)** | 13/42 **(31.0)** | 0.16 |
| Occlusion in the posterior circulation – n (%) | 6/50 **(13.7)** | 7/42 **(16.7)** | 0.52 |
| Chi² for binary variables, Kruskal-Wallis for ordinal and linear variables | | | |
